# The corona chronicles: Framing analysis of online news headlines of the COVID-19 pandemic in Italy, USA and South Africa

**DOI:** 10.4102/hsag.v27i0.1683

**Published:** 2022-02-21

**Authors:** Sumayya Ebrahim

**Affiliations:** 1 Department of Psychology, Faculty of Humanities, University of Johannesburg, Johannesburg, South Africa

**Keywords:** COVID-19 pandemic, framing analysis, health communication, media, online news, news headlines, public health

## Abstract

**Background:**

The global coronavirus disease 2019 (COVID-19) pandemic, now in its second year, has resulted in a large corpus of literature in a number of disciplines, particularly virology and epidemiology. In contrast, scholarly inquiry in other areas of the health sciences, particularly in media representations and public health communication, is still emerging.

**Aim:**

As an integral stakeholder in communication during a pandemic, this descriptive study sought to delineate the media frames of the COVID-19 pandemic in online news headlines in the first month that the COVID-19 was declared a pandemic.

**Setting:**

Online news headlines in three global hotspots, namely Italy, the USA and South Africa, during the month of March 2020, were analysed.

**Methods:**

Thematic content analysis and epidemic framing typology.

**Results:**

The findings indicate that COVID-19 has been internationally portrayed as a lethal pandemic that destroys and disrupts human life. Discursive frames of consequences monopolised its coverage, whilst discursive frames of reassurance were rare, despite the high survival rate. One of the unique findings of this study is that the COVID-19 pandemic coverage included the naming of positive patients, who were thereby made known to the public.

**Conclusion:**

Internationally, COVID-19 pandemic coverage used consequence frames that dramatised loss of life instead of deploying frames of reassurance that foreground the high survival rate of this disease.

**Contribution:**

Results of the study may help inform public health communication of the COVID-19 pandemic, by offering a detailed description of the frames that journalists use in news headlines, all of which possibly influence public perception of the pandemic. Theoretically, the article has also contributed to the application of epidemic framing typology and has contributed to knowledge in the field of public health communication and the COVID-19 pandemic.

## Introduction

Throughout the world, governments and health authorities have used media to communicate its containment and intervention strategies of the COVID-19 pandemic with its citizens. Some of the primary interventions used to contain the spread of COVID-19 pandemic have been legally enforced curfews, lockdowns, and quarantines (France-Presse 2020; Lorenzo, Jorge & Ortiz 2020). Governments have also implemented campaigns that called for social distancing and self-isolation, all of which have severely restricted people’s movement. The curtailment of in-person social interactions and the ability to socially engage with news of the pandemic centralised the role played by online news and social media in communication about the pandemic. Given the significant role of news media, the primary aim of this research, is to understand how the COVID-19 pandemic has been represented in online news headlines.

Scholars have highlighted gaps in the literature on health news coverage in the media in general (Jerit et al. 2019), and specifically there is a dearth in knowledge on news headlines (Montgomery & Feng 2016). With the objective of contributing to the empirical scholarship in the discipline of public health, specifically health communication and news frames, this study aimed to describe how COVID-19 was represented in online news headlines, specifically in the first month during which the disease was declared a pandemic.

On 11 March 2020, the World Health Organization (WHO) declared COVID-19 a global pandemic (WHO 2020a, March 11). Whilst the disease may have been first recorded in China, contagion to other regions because of international travel and subsequent local transmission have changed the virus’s foothold several times. In March 2020, Italy was the epicentre of the disease in Europe, the Republic of South Africa (RSA) in Africa, and the United States of America (USA) in the Americas (WHO 2020b, March 26). Media coverage and representations of the pandemic vary by region, based on arrival of the first case, infection, and death rates, and the impact of related consequences, such as economic cost, and/or strain on the healthcare system. Because of the global apperceptions of the pandemic promoted by the media, the role of global news in framing the pandemic proves important to interrogate, particularly as it has the potential to negatively influence public mental health.

Birnbrauer, Frohlich and Treise (2017) have recently argued that news media are likely to over-emphasise the consequences of a pandemic, such as loss of human life, but neglect to report on the aspects of its recovery and survival with the same frequency and in the same manner. Did representations at the inception the COVID-19 pandemic reflect this? How was COVID-19 represented in the first month of its status as a pandemic in online news headlines?

In response to these questions, this descriptive research aimed to characterise how the COVID-19 pandemic has been represented in online news headlines. Using framing theory as an overarching theoretical paradigm, this descriptive study aimed at identifying the news frames that were used in covering the pandemic. In particular, this study applied Shih, Wijaya and Brossard’s (2008) epidemic framing typology. These authors have identified six common frames used in the coverage of public health pandemics, namely (1) Consequence, (2) Uncertainty, (3) Action, (4) Reassurance, (5) Conflict and (6) New Evidence.

The results of the study may help inform communication regarding the COVID-19 pandemic, by offering a detailed description of the frames that journalists use in news headlines, all of which potentially influence public mental health. The findings may also assist government and public health communicators in more effectively communicating with the public about the pandemic through the media. Finally, the findings of this study will establish how news headlines in South Africa in particular compare to those generated in other global hotspots.

Having delineated the parameters of this study, the article goes on to present an outline of pandemic coverage in the media, followed by an explanation of the method and findings. This section is followed by a discussion of the results informed by epidemic framing typology. The article ends with some reflections and suggestions for future development of research regarding news media representations of the COVID-19 pandemic.

## Pandemic news coverage: Historical context

Historically, the media has played an important role in alerting the public to health crises such as the H1N1 pandemic in 2009, and the Middle East respiratory syndrome outbreak in 2012 (Maciel-Lima 2015, You & Ju 2019). The coverage tends to increase correspondingly to events such as newly identified cases, WHO announcements, governmental and health agency actions, and discoveries of new treatments or virus mutations (Shih et al. 2008; Vaughan & Tinker 2009).

News media in particular are purposeful in their communication to the public. The principle aim is to direct the public to contain the outbreak, shape attitudes, and to induce behavioural changes in the hopes of reducing mortality and morbidity (Sell et al. 2018). Mummert and Weisse (2013) argue that news media coverage has the capacity to significantly reduce the impact of a pandemic. Public engagement during a pandemic is a unique challenge, because media agents have to acquire public buy-in in order to reduce infection and death rates (Lee & Basnyat 2013).

At the beginning of the outbreak in China (November and December 2019), there was routine news coverage of COVID-19 in the global media. However, subsequent to the global spread of the virus beyond January 2020, there has been ongoing and focused news coverage across multiple platforms. Many television broadcasters dedicated 24-h news coverage to the pandemic and its associated impact on health systems, government responses, and the economic and social impact of nations’ citizens nationally and globally ( e.g. CNN, SKY NEWS and eNews Channel Africa [ENCA]). There has also been prolific engagement on social media, with millions of COVID-19 related hashtags across platforms.

Media modalities differ in the roles they play in the communication of pandemics. Social media is sometimes precarious, because of its capacity to transmit misinformation and so-called ‘fake news’ (Zarocostas 2020). At the onset of the COVID-19 outbreak, inaccurate information was in wide circulation in China (Cinelli et al. 2020; Depoux et al. 2020; Mian & Khan 2020), whilst similar reports of fake news were reported in other areas of the world (Depoux et al. 2020; Eloff 2020). The propensity of social media to create and spread fake news related to current affairs is high. Online and broadcast news, in contrast, tends to be a more credible source of accurate information.

As a sociocultural practice, health and disease communication is globally pervasive, and well-subscribed to on platforms such as Twitter and Google News, as well as in broadcast and print media (Briggs & Hallin 2016). Despite its social-cultural relevance, as an avenue of academic scrutiny, health news coverage is an area that warrants further scholarly engagement, particularly with regard to the COVID-19 pandemic. Currently, there is a modest corpus of literature on the coverage of epidemics (Lee & Basynat 2013). Evensen and Clarke (2012) and Ihekweazu (2017) have focused specifically on news coverage of infectious diseases as health crises. Jerit et al. (2019) assert, however, that there are still gaps in understanding how health crises are portrayed in the media, particularly in recognising how national and international news coverage differs in this regard. They further state that this is a crucial issue, because local media remains a key source of information in a given region.

News media in all its forms (social, broadcast, print and the web) plays an integral role in strategically communicating issues related to public health, and tends to be a source of accurate and up-to-date information (Evernsen & Clarke 2012; Viswanath & Emmons 2006). However, the news has also been a site of information which is known to increase anxiety and amplify risk perception (Strekalova 2017). The media coverage of COVID-19 as a communicable disease and the portrayal of its lethal capacity, elicit fear and anxiety, thereby compromising the mental health of the audience. For instance, Goyal et al. (2020) has reported that following the news coverage of the COVID-19 outbreak in India, a man committed suicide, believing that he was symptomatic. Nevertheless, with regard to pandemic and health crises, Sell et al. (2018) suggest that news coverage remains a fundamental aspect of strategic communication. Barry (2009) has further argued that, when managing viral public health crises, after finding a vaccine, communication is paramount.

Headlines play a particularly important role, as these draw a reader’s attention to read a news article. Headlines also assume the function of providing a summary of the news story (Montgomery & Feng 2016). According to Dor (2003), experienced readers tend to scan news headlines in order to decide what to read, and there are times when the headlines on their own provide sufficient information – to the extent that readers sometimes bypass the actual article. Despite its relevance as a source of information, there remains a dearth of scientific inquiry into headlines other than in newspapers (Montgomery & Feng 2016).

As a media practice, panic and bad news sells advertising space (Briggs & Hallin 2016). Despite this, media consumers perceive news to be fact, overlooking that the act of news reporting is itself a socially constructed process (Joye 2010), by journalists who themselves are passive actors in framing particular versions of a far from neutral story. In terms of the COVID-19 infection, the chances of survival is high, yet what the media capitalises on tends to be fatality and sensationalism.

## Method

To understand how the COVID-19 pandemic was represented in the news media, online news headlines in three countries of major global regions outside China, with the highest infection rates in the first month of the pandemic, received analysis. At the end of March 2020, these were Italy in Europe, the United States in the Americas, and South Africa in Africa.

A purposive sampling strategy was utilised in this study. Google’s search engine was used to obtain news headlines from the three countries. Google was used as it dominates global internet traffic on both mobile phones and desktops (Google Search Statistics 2020). Furthermore, Google and Google News allow search results to be customised, based on a user’s region of preference. In order to obtain a rigorous data set, the search was run three times, and each time, the preferred region was changed to Italy, then to the USA, and finally, to South Africa. News headlines with the words ‘Covid’ and or ‘Corona Virus’ were searched, as these were the most widely used terms for the illness in the public sphere. Only news headlines published in March 2020 were selected. Finally, only articles in the English language were included. Blogs and opinion pieces were excluded, as the focus was on the text and narratives used in the online news headlines. A total of (N) 814 headlines met the inclusion and exclusion criteria. Collectively, *n* = 279 were from Italy, *n* = 210 were found in the USA and *n* = 325 in RSA.

The headlines were analysed using Braun and Clarke’s (2006) six-step method of thematic content analysis. Thematic analysis allows for prominent themes and categories to be identified in a dataset (Ayres 2012). In sequence, the first step involves first becoming familiar with the data. Secondly, initial codes are generated. This study made use of one coder. Thereafter, the codes were examined to determine what potential themes could be identified. The fourth and fifth step involves reviewing the themes to determine whether they connect to the codes, followed by defining and naming the themes. The final step in the process is writing the report. Given the aim of this study, the unit of analysis was the headlines of the online news articles. As the study was focused on understanding how the COVID-19 pandemic was depicted, an inductive analytic approach was used. An inductive analysis is data driven, and does not emanate from preconceived notions. Additionally, Braun and Clarke (2006) identify two levels of interpretation, namely semantic and latent. The data was coded semantically, where priority was given to what was explicit and obvious from the headlines.

The first step was completed by copying and pasting the Google search results of the three countries into three separate documents. Each country’s headlines were read three times, and tentative notes were made. Thereafter, each headline of each country was coded. The identified codes were then stratified into possible themes and subthemes. These were then matched to the codes. Thereafter, the themes were further scrutinised and were then named and defined. The last step, as will be discussed in the following section, involved relating the identified themes to the data and existing literature.

## Results

Nine independent themes were identified from the coding process. These were as follows: (1) The deathly spread, (2) Stay home, (3) What if?, (4) The cost of COVID, (5) Treatment and Assessment, (6) Communication and Technology, (7) Government Response, (8) Face of Corona and (9) Positive News. All the themes identified are individually specific; in other words, they were unrelated to each other. The diversity of themes is evident of the distinctive representations and framings of the COVID-19 pandemic headlines. Each of these will be discussed in the following subsection.

### The deathly spread

The majority of headlines, across the three countries, reported on the number of deaths and infection more than any other COVID-related issues. For example, ‘Italy coronavirus deaths at 5476 after 651 rise’ (Mayberry, Alsaafin & Pietromarchi 2020) and ‘First Covid-19 (sic) deaths in South Africa announced as infection toll reaches 1000’ (Ellis 2020). Majority of the headlines were expressed using figures to denote the number of cases. In addition, numerical milestones were headlined, such as the 10 000 mark. The highest number of deaths was recorded in Italy during March 2020, as the pandemic was already widespread there. In contrast, South African and USA headlines drew attention to the number of firsts and locations of infections, as these countries were behind Italy in terms of the course of the pandemic.

### Stay home

Coded items that encompassed the restriction of movement included headlines referring to lockdowns, quarantine, travel and social bans, as well as the closure of national borders. As responses to the pandemic evolved, one of the catchphrases in communicating its containment was ‘stay home’. This theme also included reference to passengers who were stranded on ships, as nations were not allowing seafarers into their countries at their sea borders. Headlines emphasising governmentally instituted restrictions of movement were featured globally, and were widely implemented as a strategy to contain COVID-19 infections. An instance of this is: ‘Don’t travel, don’t socialise, stay inside: Italy’s coronavirus lockdown rules’ (France-Presse 2020). Similarly, ‘“Stay Home, Stay Healthy”: These states have ordered residents to avoid nonessential travel amid coronavirus’ was a headline that appeared in USA Today (Lorenzo et al. 2020).

### What if?

During analysis, headlines that indicated future projections and speculation of the pandemic were coded. Headlines such as warnings of what could happen, hypothetical situations, estimations, expectations, and predictions were frequent in all three national news headlines. Speculation in Italy was evident in the headline ‘Italy’s Nightmare Offers a Chilling Preview of What’s Coming’ (Silver, Migliaccio & Follain 2020) ‘Africa faces grave risks as COVID-19 emerges, says Berkeley economist’ (Lempinen 2020) and ‘Too many coronavirus patients, too few ventilators: Outlook in US could get bad, quickly’ (Alltucker & Penzenstadler 2020), are indicators of this theme. This was particularly frequent in USA news headlines. The next prominent theme that was identified was economic and finance matters owing to the COVID-19 pandemic.

### The cost of COVID

The economic and financial costs of the COVID-19 pandemic were evident in the headlines. Many headlines featured issues related to pecuniary matters on national and international levels, such as government aid to fight COVID-19, or leaders borrowing money from others to contain the pandemic within their own countries. ‘The 25-billion-euro plan to rescue Italy’s economy from coronavirus crisis’ (The Local Italy 2020) is but one example. There were also numerous references to the economic impact of the pandemic on individual entities and private businesses, as well as the financial rescue options available to them (Business Insider 2020a; Rajgopal 2020). Headlines pertaining to the economic impact were particularly relevant in South Africa, such as ‘Small businesses in SA can now register for help during the coronavirus disaster’ (Business Insider 2020b). The next theme that emanated from the coding was the treatment and assessment of COVID-19.

### Treatment and assessment

As is common in pandemic reporting, a number of headlines were coded that referred to the assessment and treatment of COVID-19. In this regard, headlines made mention of vaccines, drug trials, as well as testing and strategies geared towards heard immunity as methods of containing the pandemic. The majority of these were evident in news articles from the USA and RSA. This was expected, as these countries were still strategising around assessment and treatment plans. For instance, ‘Search for Coronavirus Vaccine Becomes a Global Competition’ (Sanger et al. 2020). In RSA, headlines referring to testing was common place. One headline read: ‘Covid-19 test available to South African public from today’ (Le Roux 2020). The next theme was media communication and technology.

### Communication and technology

Items relating to communication and technology were coded from the data. These codes contributed to a theme that covered the use of communication and technology and its role in communication of the pandemic. This featured the use of WhatsApp and Facebook as tools to communicate pandemic information and updates to the public. For example, Morais (2020) reported ‘Coronavirus: 1.5 million people use Covid-19 WhatsApp helpline in under a week’. Headlines also featured the role of new technologies, such as 3D printing and computer programmes to combat the pandemic. Headlines that described the problem of fake news were also coded from the data set. This featured prominently in the South African media, where the act of spreading fake news was criminalised, as depicted in the following headline: ‘COVID-19: 6 months in jail for publishing fake news’ (Eloff 2020). The next theme was government responses to the COVID-19 pandemic.

### Government response

National government responses and those of political parties in all three countries were headlined in online news. These centred on communicating various national updates and responses to the pandemic. This theme was consistent throughout the three countries. ‘U.S. becomes first country to report 100 000 confirmed coronavirus cases; Trump invokes Defense Production Act’ (Iati et al. 2020) and ‘Coronavirus: National state of disaster takes effect with publishing of gazetted regulations’ (Karrim 2020) illustrate this. In Italy, the comment from the Prime Minister was headlined as follows; ‘Italy’s lockdown will be extended, prime minister says as death toll spikes and hospitals struggle’ (Ellyatt 2020). Following government responses was a theme that pertained to the personification of the COVID-19 pandemic.

### Face of corona

Unexpected findings in the analysis saw headlines declaring the health status of public personalities testing either positive or negative for the virus. Many journalists put a human name and face to the disease of well-known international personalities across all three countries. At the early stages of the pandemic, Hollywood actors Tom Hanks and Rita Wilson, British Prime Minister Boris Johnson, and Prince Charles of England were amongst a few public figures reported to have tested positive for COVID-19. The pattern of naming was more prominent in RSA and Italy, than in the USA. ‘Juventus footballer Daniele Rugani tests positive for coronavirus’ (Aljazeera 2020) and ‘ACDP leader Kenneth Meshoe tests positive for Covid-19’ (Mahlati 2020), are some examples. The practice of personifying the pandemic is uncommon, and seems to be a unique feature of the representations of the COVID-19 pandemic.

### Positive news

One of the themes that had the fewest meaningful codes was that of positive news. However, in thematic analysis, Braun and Clarke (2006) suggest that items that are not part of a dominant story also merit coding and inclusion. These headlines related to any positive progress or hopeful news of the pandemic, such as a drop in infection rates, decline in cases, and or recovery of patients. Headlines of this sort were only recorded in Italian and RSA news. For example ‘Coronavirus cure hope as Italian man, 79, recovers after taking experimental Ebola drug …’ and ‘Coronavirus: SA’s Patient Zero recovers’ (ENCA 2020). In March 2020, no positive news was noted in the selected USA headlines.

Having outlined the emergent themes, the section that follows offers a discussion of these findings.

## Discussion

News headlines are a powerful mode of media representations that portray events in specific ways (Joye 2010). Moreover, news frames select some aspects of a perceived reality and make them more salient. The effect of this is that a particular definition and understanding of the problem is promoted. To elucidate the themes that were identified, Shih et al.’s (2008) epidemic framing typology is drawn on: (1) Consequence, (2) Uncertainty, (3) Action, (4) Reassurance, (5) Conflict and (6) New Evidence.

Five of the six identified frames were found in the current data set. One frame referring to conflict (differences of opinions and arguments about the disease) was not identified. The COVID-19 virus is a novel pathogen and virologists and scientists are still learning about the virus. Therefore, conflict frames were not prominent in the March 2020 online news headlines as yet. However, a cursory view of headlines after March 2020 indicates that these could manifest as research outputs increase. The five remaining frames will be discussed below.

The first and most salient frames identified in the headlines were that of *consequence*. The themes of Deathly Spread, Stay Home and The Cost of COVID fall within the consequence frame. Consequences are understood as the cost and effects of the disease on humanity such as sickness and death, and the social, economic and political impacts (Shih et al. 2008). Consistent with Birnbrauer et al. (2017) this study has revealed that headlines that centralised the loss of human life were the most dominant frame in the early months of COVID-19 pandemic coverage. According to Joye (2010), it is common for news coverage to be proportionate to the number of people who succumb to disease as opposed to the number who have survived and recovered. The high recovery rate of COVID-19 was rarely reflected in the headlines, as it is standard journalistic practice to report primarily on the devastation that pandemics bring to humanity. Previous inquiry by Maciel-Lima et al. (2015) showed how daily reports of the H1N1 pandemic would constantly include statistics on the number of cases registered, victims, hospitalisation and death rates, both nationally and globally. For the current COVID-19 pandemic, in addition to news headlines, there are dedicated websites tallying the cost on human life in real time such as follows: https://www.worldometers.info/coronavirus/ and Live Status Report COVID-19 (https://app.powerbi.com/).

Other consequences emphasised in headlines were restrictions of people’s movement and the global economic impact that the pandemic has caused. This included how governments financially invested in fighting the pandemic, as well as how governments offered financial assistance to individuals and businesses impacted by the pandemic (Business Insider 2020b; The Local Italy 2020). Collectively, these headlines focused on the spectacle and damage that the COVID-19 pandemic has caused to contemporary life and the alarming tone of the headlines is likely to evoke fear and anxiety in its audiences.

Secondly, headlines that featured *uncertainty* frames were prominent in the sample. These were coded under the theme of ‘What if?’ This frame refers to the indeterminate aspects of the disease (Shih et al. 2008) such as the potential impact it may have on a city’s population, how many people could actually die and what potential impact this could have on humanity and the economy. Virologists and scientists are still learning about the virus, yet media houses prematurely speculated about the potential health impact, all of which fuel what would not be an exaggeration in this instance to call, public pandemonium (Mian & Khan 2020). Headlines such as these serve not only to illustrate the amount of gratuitous distress they can create, but also highlight how news media is more than mere reporting of facts. In this regard, headlines were framed as possible projections and estimates of the disease. Once again, these speculative headlines capitalised on the dramatic and anxiety-provoking strategies used by journalists when covering unknowns about pandemics. The possible effect is that this could elevate feelings of anxiety and fear in the public, but equally also draw attention to the headlines by stimulating alarm and distress, all of which potentially compromise the mental state of the reader.

The third frame that was identified was an *action* frame. This frame pertains to actions taken by authorities and governments against the disease (Shih et al. 2008). The themes of Communication and Technology and Government Responses fall under this frame. Health authorities, together with national leadership, take a central role in managing pandemics (Lee & Basynat 2013). Actions frames were a key feature in headlines and included references to governmentally instituted restrictions of movement (such as lockdowns and quarantines) as well as government financial responses to the pandemic (such as financial investment into managing the pandemic). The theme of communication and technology also fit into the action frame as it pertains to what action and measures leaders have taken in response to the pandemic.

*Reassurance* was the fourth frame recognised from the analysis. Headlines coded under the theme of Positive News are captured by the reassurance frame. Reassurance frames are those that emphasise success or triumphs in the pandemic. Frames of reassurance are rare in pandemic and public health crisis reporting and were expectedly the least used frame in the analysis. Although the majority of COVID-19 cases recover, the headlines did not reflect this. Instead, attention was repeatedly paid to the number of people who have succumbed to the virus, or who have been infected by it. The tradition of selling bad news is well established in media practice (Arango-Kure, Garz & Rott 2014). Consequently, emphasis on recovery will deter from the broader agenda of reporting on crises and drama of the pandemic. Moreover, reporting of positive news also carries the hazard of people not taking the pandemic risks seriously, as risk frames seem to be the preferred frame in pandemic communication and containment.

The final frame that was identified was *new evidence*. This frame characterises the results of scientific and scholarly progress towards containing, curing, and preventing the disease (Shih et al. 2008). The theme of Treatment and Assessment can be understood by the New Evidence frame. Numerous headlines referred to the drugs, vaccines, and assessment methods. This type of framing is expected in new pandemics. These frames play an important role in keeping audiences informed on the latest developments that could treat and or prevent infection and thereby curb the spread of the pandemic.

The identified typologies were in keeping with frames used in reporting public health crises and pandemics in the past. In March 2020 online news headlines, the massive human cost of the pandemic informed the majority of news frames covering the COVID-19 pandemic. In health communication, negative emotions are usually drawn on to mediate responses. Negative emotions such as fear underlie the strategies to persuade people to act in accordance with health authority directives to contain pandemics.

Whilst the majority of the news frames were similar across all three regions, there were some differences. Each of the countries selected were in a different stage of the COVID-19 pandemic in March 2020. The differences in headlines reflect the phase at which each nation was at in the pandemic course. Consequence frames of death were mostly in Italy, as they were well into the pandemic, whereas, consequence frames in the USA and South Africa were based on infection rates, as the pandemic had not yet peaked. All three countries made use of Consequence, Uncertainty, Action, and New Evidence Frames, whilst Reassurance frames were only found in Italy and South Africa. As mentioned earlier, the conflict frame was not found in the analysis.

A unique finding in this study was the naming of people who have tested positive for the virus. Unlike in coverage of other pandemics, journalists covering the COVID-19 pandemic have routinely named public figures who had tested positive for the virus. Details were reported on public personalities as well as their journey through to recovery or death. This practice does not precisely fit into the frames identified by Shih et al. (2008). One of the most plausible explanations of personifying the virus is to establish rapport with the readers and to demonstrate how equally vulnerable all members of society are to the virus.

## Strengths and limitations

A strength of this study was the inductive method of coding and framing. The inductive approach allowed the data to lead the findings and allowed for the frames to be developed from the data organically. An additional strength of the study was that in selecting the three global regions, headlines that were circulating through different phases of the pandemic at the same point in time were included in the analysis. A recommendation for improving the study would be to repeat the study with an additional coder and to include a quantitative content analysis to gauge statistical significance of the findings. A further weakness was that only headlines in the English language were used in the data set. This study was also limited in terms of the inferences that could be made regarding the impact of these headlines on the public. Future researcher targeted at reader’s subjective experiences of the COVID-19 pandemic news coverage will augment the scholarship on how the public have actually perceived the news coverage and how this exposure may have affected subjective the mental health of the readers.

## Conclusion

News media play an integral role in communication during a pandemic, and a descriptive analysis of the representations at the outset of the pandemic had yet to be empirically investigated. This study contributes to the empirical scholarship in the discipline of public health of the COVID-19 pandemic, specifically health communication and news frames. Using framing theory, specifically epidemic framing typology, as an explanatory model, this study further aimed to identify the news frames that were used in covering the pandemic.

Fundamentally, this study has shown that internationally, predictable consequence news frames dominated the pandemic coverage in the first month of it being declared. Wald (2008) records that there is both fear and fascination with pandemic outbreaks in academia and mainstream media. This fear and fascination seems to underlie much of what is reported on pandemics. Consistent with Wald (2008) international COVID-19 pandemic coverage used consequence frames that dramatised the loss of life and the impact on daily human life.

This study has contributed to the application of epidemic framing typology and public health communication. Moreover, it has contributed to the emerging COVID-19 scholarship as it is located at the intersection of online news headlines and public health communication. It has also contributed to scholarly work in the South African context with regard to public health communication and COVID-19.

It seems that in an attempt to increase media consumption, media organisations routinely capitalise on dramatic headlines in their coverage of the COVID-19 pandemic, thereby inducing panic amongst the public, despite the high survival rate. The pandemic continues to have far-reaching individual, societal, national, and global consequences. However, long after the pandemic ends, the news headlines of the pandemic will concretise the way in which it gets recorded for posterity, as well as influence public memory of this historic event.
